# Assessment of network structure characteristics and factors of corporate flows in Guangdong Province

**DOI:** 10.1371/journal.pone.0293870

**Published:** 2024-03-08

**Authors:** Xuejiao Chen, Yong He, Teng Long, Junxiu Wang, Xueye Chen

**Affiliations:** 1 School of Architecture&Urban Planning, ChongQing Jiaotong University, Chongqing, China; 2 Piesat Information Technology Co., Ltd., China; 3 Key Laboratory of Urban Land Resources Monitoring and Simulation, Ministry of Natura Resources, Shenzhen, China; Northeastern University (Shenyang China), CHINA

## Abstract

With the rapid development of the world city network, the traditional location theory has gradually been disproven, and the advantages of the flow space over the traditional vertical organizational structure are gradually being revealed. Therefore, from corporate branch networks and corporate investment networks, 21 cities in urban agglomerations of Guangdong are taken as case studies for this paper. Furthermore, in this paper, 5 representative types of corporate contact data (catering service, financial service, life service, sports and leisure and accommodation service) are selected, the social network analysis (SNA) method is used to quantitatively analyze the network structure characteristics of urban agglomerations, and a spatial interaction model is constructed to explore the factors influencing. The results indicate that secondary networks have developed in Guangdong. The financial service network is the most complex, followed by the life services, sports and leisure and catering networks. The accommodation service network structure is the simplest. Among all kinds of networks, Guangzhou and Shenzhen have the highest status. The catering and accommodation corporations in Yangjiang in the west have a relatively major external development. Shantou in the east has many branches of various types, while most of the capital exchange in the region is concentrated in Heyuan and Qingyuan in the north. The coefficients of geographical proximity and the urban development level play a significant role in promoting the development of networks. However, administrative capacity limits the attractiveness of origin cities to a certain extent.

## 1. Introduction

At the end of the 20th century, with the rapid worldwide expansion of multinational corporations, a new regional division of labor, global value chains, global commodity chains, global production networks and new trade theories gradually emerged [1, 2]. With the rapid development of information technology and modern science and technology, the cost of space and time distance has been continuously compressed, and the pace of globalization has also moved forward [3]. The flows of people, capital, consciousness and information around the world are intricately intertwined [4].

Against the background of globalization and informatization, the concept of "flow space" was proposed. It was believed that the traditional society would eventually transform into the economic and spatial structure of information society [5]. Currently, the ruling form of social organizations has gradually changed from a vertically integrated class organization to a network form of organization [6]. Global cities are the original driving force of flow space [7]. Interaction between cities diminishes the spatial distance between geographical entities and forms an urban network with an apparent spatial structure and organizational function [8]. With a deepening understanding of cities, the spatial structure of urban agglomerations has become one of the leading research topics of urban geography [9]. The research perspective has also shifted from the development of a single city to an urban network. Moreover, there is a focus on the characteristics, structure, function and relationship of the spatial structure of urban network based on relational data [9–13].

In the early stage, the bottleneck in the study of the global urban network was the lack of relational data. Most of the data obtained were at the national level, while the city-level data were difficult to obtain. In general, the demographic, economic and other attribute data obtained from statistical methods can only measure the important characteristics of cities, but cannot explain the correlation characteristics and spatial patterns between cities [14]. In the 21st century, the Globalization and World Cities Study Group and Network (GaWC) overcame the original research limitations. It used a variety of relational data, such as infrastructure, transnational migration, and corporate contacts, to study the world urban network. Since then, research on the world urban network has entered a new stage.

Connection between cities are considered to be their second nature. The essence of urban networks is the connection between cities, and cities are the initial driving force of mobile space [15]. Therefore, the chain network model was proposed and has made outstanding contributions to the research of urban networks, supported the transformation from corporate connections to urban relationships, and provided a new method for the research of urban networks [16].

Later, improved algorithm based on the interlocking network model was proposed. For example, recursive centrality and recursive force are proposed to measure the status of cities [17]. In addition, inspired by this model, algorithms such as zoning and directed weighting analysis (DWD) for urban networks have been proposed [18], as well as models such as the agent-based urban simulation model (ABUSM) [19]. These models and methods still focus on macroindicators, such as node power and node connection structure. With the social network method, the division of functional blocks and microinfluencing factors are further studied.

The social network analysis method is widely used in urban network research. This method was first used in a global urban network study in 2004 [20]. The concepts of centrality, degree centralization (DCZ) and core edges could help us understand urban networks. This method is also commonly used today.

Social network analysis data include infrastructure, human activity trajectories, social and cultural data, and innovation connectivity data. When company organizational data are difficult to obtain, infrastructure data are the primary information source for studying the world urban network. Air passenger flow, road passenger flow, port cargo volume, and internet hardware facility data, are relatively common infrastructure data, that can be used to study the relationships and connections among world cities [21], national cities [22], port cities [23], and even urban functional areas [24]. Therefore, urban network research based on infrastructure pathway involves national and regional levels.

Commuter data are the most commonly used human activity trajectory data. In recent years, the use of crowd activity trajectory data has increased. The data are often provided by internet-related institutions or crawled by individuals from websites [25]. There are many studies on people’s intercity mobility based on big data, such as mobile signaling, microblog check-ins and the Baidu index [26]. In addition, human activity trajectory data based on shopping, leisure and business travel are commonly used. Representative studies include multicenter urban areas [27].

Social and cultural data represent a set of people who express human perception and consciousness. This research based on social and cultural data focuses on the urban network that is formed through the promotion of social, cultural, political and other factors. In recent years, with the emergence of new network media and changes in people’s consumption views, the research on urban networks based on network social space and cultural consumption space has attracted widespread attention. Shared access network data can be used to study the characteristics of urban networks [28]. The cooperation patent data of cities and cross-regional cooperation patent data can be used to measure the efficiency of knowledge innovation [29] and study the urban innovation networks [30].

After years of exploration, global urban network research has produced rich results. Currently, many studies are not limited to a specific type of data or the abovementioned methods, but focus on big data from multiple sources. On the whole, the research content of urban networks has experienced a transformation from the attributes of cities to relationships between cities, the research basis has experienced a transformation from a central place to flow space, the research data have experienced a shift from "spatial interaction model" relationship simulation to the capture of accurate relationship data between cities, and the research elements have experienced a transformation from "hard" to "soft" networks. The research vision has experienced multiple changes from focusing on a single scale to paying attention to different scales, such as global, regional and national scales, making urban networks an important research paradigm for interpreting urban relations and urban spatial organization. In terms of research methodology, multifaceted approaches such as the spatial interaction model, chain network model, headquarters branching model and social network model also have their own focuses in view of the different characteristics of urban networks.

**Fig 1:**
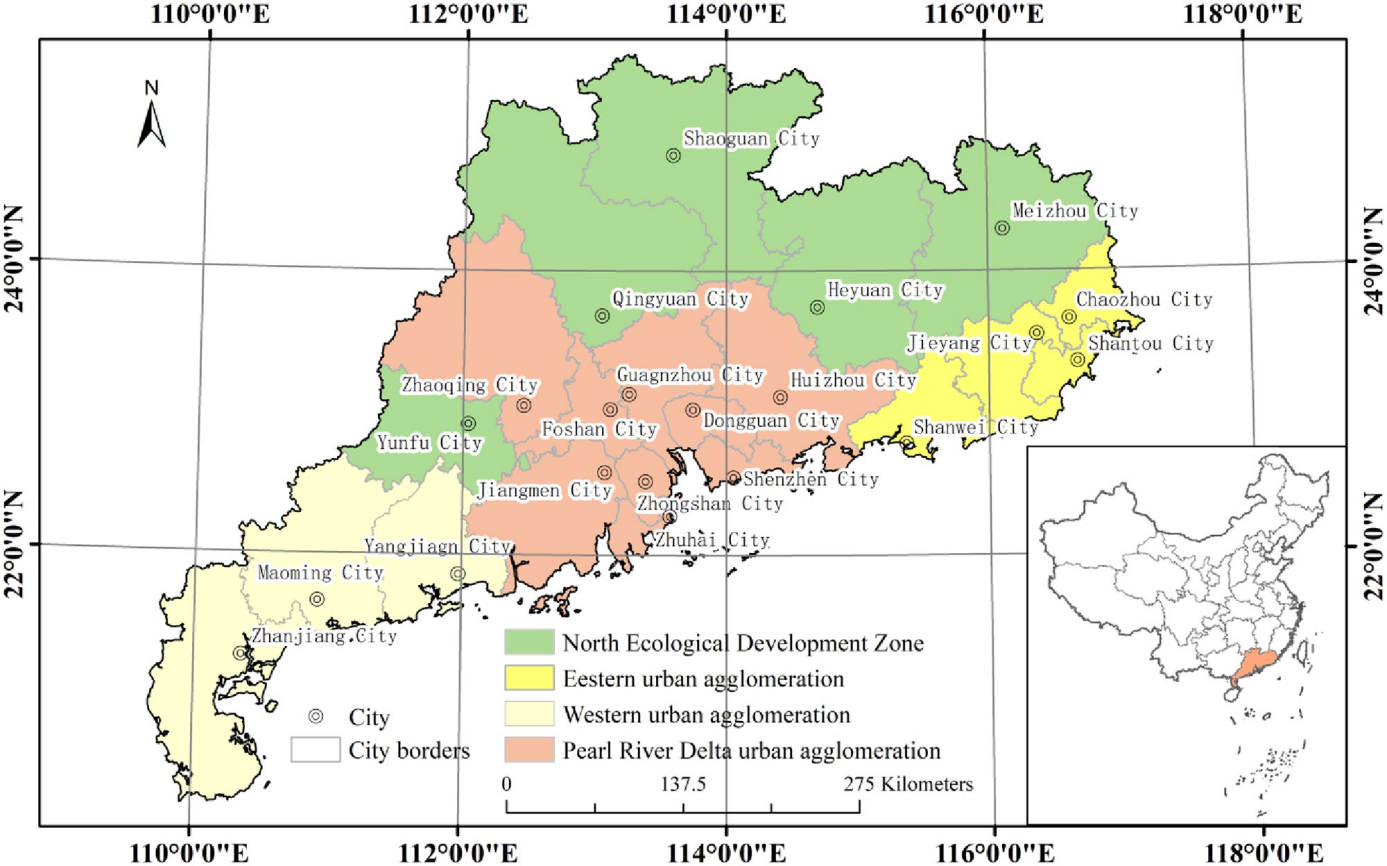
Study area of Guangdong Province. Note: Fig 1 was created based on the Ministry of Natural Resources’ standard map service website (http://bzdt.ch.mnr.gov.cn/) No. GS(2020)4619 map, base map boundary not modified.

To identify the development impact and characteristics of world-class cities on regional urban networks, Guangdong Province of China (Fig 1) is selected as the research area for this paper. Because the world-famous Guangdong-Hong Kong-Macao Greater Bay Area is part of Guangdong Province of China, and China’s unique political system makes independent provinces generally closely connected and develop together, and the impact of large cities on regional networks will be more obvious. Its network structure is more complex and mature than the general regional structure, which is helpful for discovering the deep characteristics of the regional network under the gradual development of the world urban network, which may not be available in other networks.

## 2. Datasets

1. Five kinds of enterprise contact data. The data used in this paper are corporate flow data from Shenzhen Weihe Technology Co., Ltd. (http://www.wayhe.com/), including corporate investment data and headquarters and branches data in 2018. The data level is aggregated to cities. The primary data of this paper are the statistics of the distribution and capital exchange among all enterprises in the prefecture-level city and enterprises in other prefecture-level cities. The flow data are CSV data, including those of the origin city (the place where funds flow out, or the head corporation) and the destination city (where funds are transferred in or the subsidiary corporation), and the information and types of flow data are shown in Table 1. Catering service is an indicator of quality of people’s lives and includes enterprises that provide people with various drinks, food, consumption places and facilities. Sports and leisure are indicators of the quality of people’s leisure time, including cultural, sports and entertainment enterprises. Financial service refers to a particular industry that is closely related to a city’s development and operates financial commodities, including banking, insurance, trust, securities, and rental enterprises. The life service industry is an important indicator of people’s living standards, including housekeeping, washing and dyeing, bathing, beauty salons, home appliance maintenance, portrait photography, and other enterprises. Accommodation service is an indicator of personnel mobility and includes corporations such as hotels, lodges, inns and resorts.
2. Attribute data. The factors influencing are selected from the China Urban Statistical Yearbook, with a total of 21 cities. Considering the lags and contingency of the development of cities, the average value from 2014 to 2018 is adopted for the indicator (https://data.cnki.net/ Yearbook).

**Table 1:** Corporate data sheet unit: Corporate investment flow (×104 million yuan); corporate branch flow (PCs); proportion (%).

Category	numbers	corporate investment				corporate branch			
		out quantity	proportion	input quantity	proportion	output quantity	proportion	input quantity	proportion
Catering	42733	817	0.56	744	0.93	218	14.04	321	21.96
Sports and leisure	26404	3538	2.43	5055	6.33	169	10.88	135	9.23
Finance	6121	132473	91.03	68725	86.00	778	50.10	584	39.95
Life service	18431	8306	5.71	2097	2.62	367	23.63	380	25.99
Accommodation	3201	395	0.27	3287	4.11	21	1.35	42	2.87
Total	96890	145529	100	79909	100	1553	100	1462	100

## 3. Methodology

### 3.1 Network centrality

The social network analysis method is a mature research method in social science fields. It comprehensively uses graph theory, matrices and other models to study the relationship and network structure between different urban nodes. This study analyses the node attribute characteristics, connection patterns and overall network characteristics of urban agglomeration networks from multiple perspectives.

#### 3.1.1 Degree centrality and degree centralization

**Fig 2:**
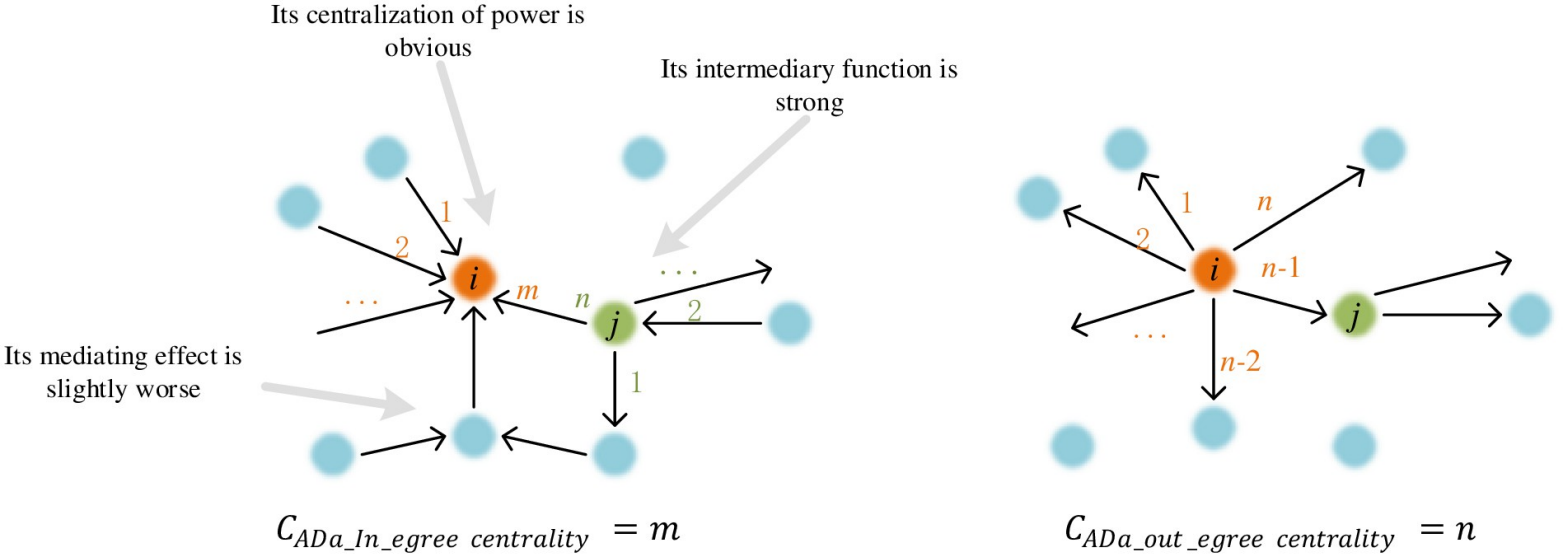
Schematic diagram of point centrality.

Degree centrality (DC) is used to measure the connection degree between a node and other nodes. If a node is directly connected with many other nodes, then it is considered to have a high DC. In a directed network (Fig 2), the DC can be divided into in-DC and out-DC. The in-DC is the number of other nodes entering the node, that is, the number of direct relationships obtained by the node, and the out-DC is the number of relationships directly sent by the node. In this paper, DC does not include indirectly connected nodes; thus, it is "local centrality", and the ranking between nodes is more noteworthy.

DCZ can represent the tendency of a network to concentrate on one node, so the degree of centralization in the overall network can be measured. First, we calculate the difference between the theoretical maximum centrality and the actual centrality of each node in the network. Then, calculate the sum of the difference between the former. Finally, divide the sum by the sum of the difference. The formula is:

$$C_{AD}=\frac{\sum_{i=1}^{n}\left(C_{ADmax}-C_{ADi}\right)}{max[\;\sum_{i=1}^{n}\left(C_{ADmax}-C_{ADi}\right)]}$$ (1)

where *C*_*ADi*_ is the centrality of node *i* and *C*_*ADmax*_ is the theoretical maximum DCZ. The greater the DCZ is, the more concentrated the connections in the network are in fewer nodes and the more obvious the network centralization characteristics are.

#### 3.1.2 Betweenness centrality

In the urban network, if connections among multiple cities must pass through a city, that city can be considered to have an important position [31]. The index describing individual centrality is betweenness centrality (BC), which reflects the degree of individual control over resources [32]. Numerically, the closer the BC of a node is to 0, the fewer nodes it can control, the weaker its intermediary role in the network, and the closer it is to the edge of the network. In contrast, the higher the value is, the greater the power of the node, the stronger the intermediary role, and the closer it is to the core position of the network.

Suppose the number of links between node *j* and node *k* is *g*_*jk*_. Then, the number of links passing through the third node *i* between node *j* and node *k* is *g*_*jk*_ and the ability of the third node *i* to control the communication between node *j* and node *k* is. That is, the probability that node *i* is on the link between node *j* and node *k*, is *b*_*jk*_ (*i*). The formula is:

$$b_{jk}\left(i\right)=\frac{g_{jk}\left(i\right)}{g_{jk}}$$ (2)

The centrality of all node pairs of node *i* in the network is added to obtain the BC of the node, *C*_*ABi*_. The formula is:

$$C_{ABi}=\Sigma_{j}^{n}\Sigma_{k}^{n}\;b_{jk}\left(i\right)\;j\neq k\neq i,且j<k$$ (3)

#### 3.1.3 Network density

In this paper, the network density refers to the ratio of the number of city nodes connected in the city network to the maximum number of theoretically possible city connections. In an undirected network with *n* nodes, the theoretical maximum of the total number is:

$$n\left(n-1\right)/2$$ (4)

Suppose the actual number of relationships in the network is *m*. In this case, the density is the ratio of the actual number of relationships to the theoretical maximum number of relationships, that is:

$$m/\left(n\left(n-1\right)/2\right)=2m/\left(n\left(n-1\right)\right)$$ (5)

The higher the value is, the closer the connection between the networks. In this study, the network is a directed network. The matrix data of cities are binarized, the connected cities are assigned a value of 1, and the unconnected cities are assigned a value of 0. The density is an indicator of the tightness of the connection between nodes, and its value is between [0,1]. The closer the value is to 1, the closer the connection between nodes and the higher the level of development of network.

### 3.2 Spatial interaction model

#### 3.2.1 Selection of model indicators

Capital flows and branch structure between enterprises can reflect the closeness of economic ties in cities as well as the competitiveness and influence in regions. The exchange of capital and the establishment of branches in cities are the internal factors driving the composition of urban networks. Enterprises realize their development by establishing branches and exchanging capital with other regions, and they also participate in the construction of remote urban networks. In this process, the vitality of local cities supports the development of a corporate network structure, while the choice of location is related to the attractiveness of converging urban attributes, and the cost of the space-time distance between the two places is a constant source of resistance in the development of the entire urban network.

Urban resource endowment, location conditions, economic level, policies and measures, space-time distance and other factors jointly affect the regional network structure. The higher the economic level of the city is, the larger the population and the size of the city. The stronger the core engine power is, the stronger the urban vitality and the higher the economic demand. Such a city has a strong attraction to the outside and a strong aggregation capacity to the inside. The number of urban enterprises, scientific and technical personnel and scientific research institutions is important for supporting corporate development, which can ensure not only that local enterprises have sufficient technical support but also that foreign enterprises can achieve long-term and stable development. Spatiotemporal distance is another important factor influencing regional development in location theory. Currently, due to the rapid development of transportation, the space-time distance has been greatly compressed, but it is still an important factor that should be considered. In addition, regional barriers are obvious in the urban development system with Chinese characteristics, and the political system plays an important role [33].

Considering the availability and scientific city of various indexes, the selection and meaning of indexes are shown in Table 2. In addition, to avoid excessive numerical differences between the indicators, most indicators are counted per population unit.

**Table 2:** Description of corporate factors influencing and indicators.

index	explanation	units
Urban development level	Per capita GDP	Ten thousand yuan
Urban enterprise vitality	Number of industrial enterprises per 10000 persons	PCs/km^2^
City scale	Urban construction land area	km^2^
Urban educational resource reserve	Number of ordinary colleges and universities per 10000 persons	PCs/km^2^
Urban market demand	Annual average population	Ten thousand people
Urban administrative capacity	The administrative level of the city is 4 for the provincial capital city, 3 for a sub provincial city, 2 for a special economic zone, and 1 for the rest	/
Reserve of urban technicians	Number of scientific research, technical service and geological survey personnel per 10000 persons	People/km^2^
Urban opening level	Amount of foreign capital actually used per 10000 persons	Ten thousand dollars
Urban science and technology support	Science and technology expenditure per 10000 persons	Ten thousand yuan
Geographical proximity	The greater the distance of enterprise communication in geospatial terms, the closer the geographical proximity	/

Due to the proximity effect of geography, the influence effect of cities tends to decrease with increasing space-time distance. Although the network of capital exchange between cities is a virtual network, it is still affected by space-time distance. Given the simplicity of the index, the Euclidean distance between cities is used in this paper to measure the space-time distance. Considering that the actual distances will differ greatly, the index is processed according to Formula 6:

$$Geodis_{ij}=1-\text{ln}\left(\frac{d_{ij}}{maxd_{ij}}\right)$$ (6)

where *Geodis*_*ij*_ is geographical proximity, and the higher the value is, the closer the geographical distance, *d*_*ij*_ is the Euclidean distance from city *i* to city *j*, and *maxd*_*ij*_ is the largest Euclidean distance in the index.

#### 3.2.2 Construction of the model

To explore the factors influencing urban networks, this paper constructs 3 subsystems, origin city vitality, destination city attractiveness and geographical proximity, and considers the impact of indicators on corporate mutual investment and branches.

Because the dependent variable has a value of 0 and the data are too scattered, it is difficult to achieve a normal distribution, and traditional linear regression is difficult to apply. Therefore, this paper uses the negative binomial regression model to explore the factors influencing of corporate mutual investment and branches. The regression model of factors influencing of corporate mutual investment is Formula 7, and the review model of the factors influencing of headquarters and branches is Formula 8:

$$\begin{array}{c} Y_{ij1}=\alpha_{1}VitGDP_{i}+\alpha_{2}Vitpeo_{i}+\alpha_{3}Vitcon_{i}+\alpha_{4}Vitent_{i}+\alpha_{5}Vitedu_{i}+\\ \beta_{1}AttGDP_{j}+\beta_{2}Attpeo_{j}+\beta_{3}Attent_{j}+\beta_{4}Attpub_{j}+\beta_{5}Attsci_{j}+\beta_{6}Attentfor_{j}+\\ \beta_{7}Attexp_{j}+\gamma Geodis_{j}+\varepsilon_{ij}+z_{0} \end{array}$$ (7)

$$\begin{array}{c} Y_{ij2}=\alpha_{1}VitGDP_{i}+\alpha_{2}Vitpeo_{i}+\alpha_{3}Vitsci_{i}+\alpha_{4}Vitent_{i}+\alpha_{5}Vitedu_{i}+\alpha_{6}Vitfor_{i}+\\ \beta_{1}AttGDP_{j}+\beta_{2}Attpeo_{j}+\beta_{3}Attent_{j}+\beta_{4}Attpub_{j}+\beta_{5}Attsci_{j}+\beta_{6}Attentfor_{j}+\\ \beta_{7}Attexp_{j}+\beta_{8}Attentfor_{j}+\beta_{9}Attedu_{j}+Geodis_{j}+\varepsilon_{ij}+z_{0} \end{array}$$ (8)

where *Y*_*ij*1_ is the amount of capital exchange between city *i* and city *j*. *Y*_*ij*2_ is the number of branches from city *i* to city *j*, both of which are vectors. *Vit* is the origin city vitality subsystem. *Att* is the destination city attractiveness subsystem. *GDP* is per capita GDP. *peo* is the annual average population. *con* is the number of industrial enterprises. *ent* is the area of urban construction land. *edu* is the number of ordinary colleges and universities. *pub* is the urban administrative level. *exp* is science and technology expenditure. *for* is the actual amount of foreign capital used where *i* is the origin city and *j* is the remitting city. *Geodis* is the geographical proximity between cities. *ε*_*ij*_ is the error term, and *z*_0_ is a constant term.

## 4. Results

### 4.1 Analysis of network centrality

#### 4.1.1 Quantitative results analysis of the DC

**Fig 3:**
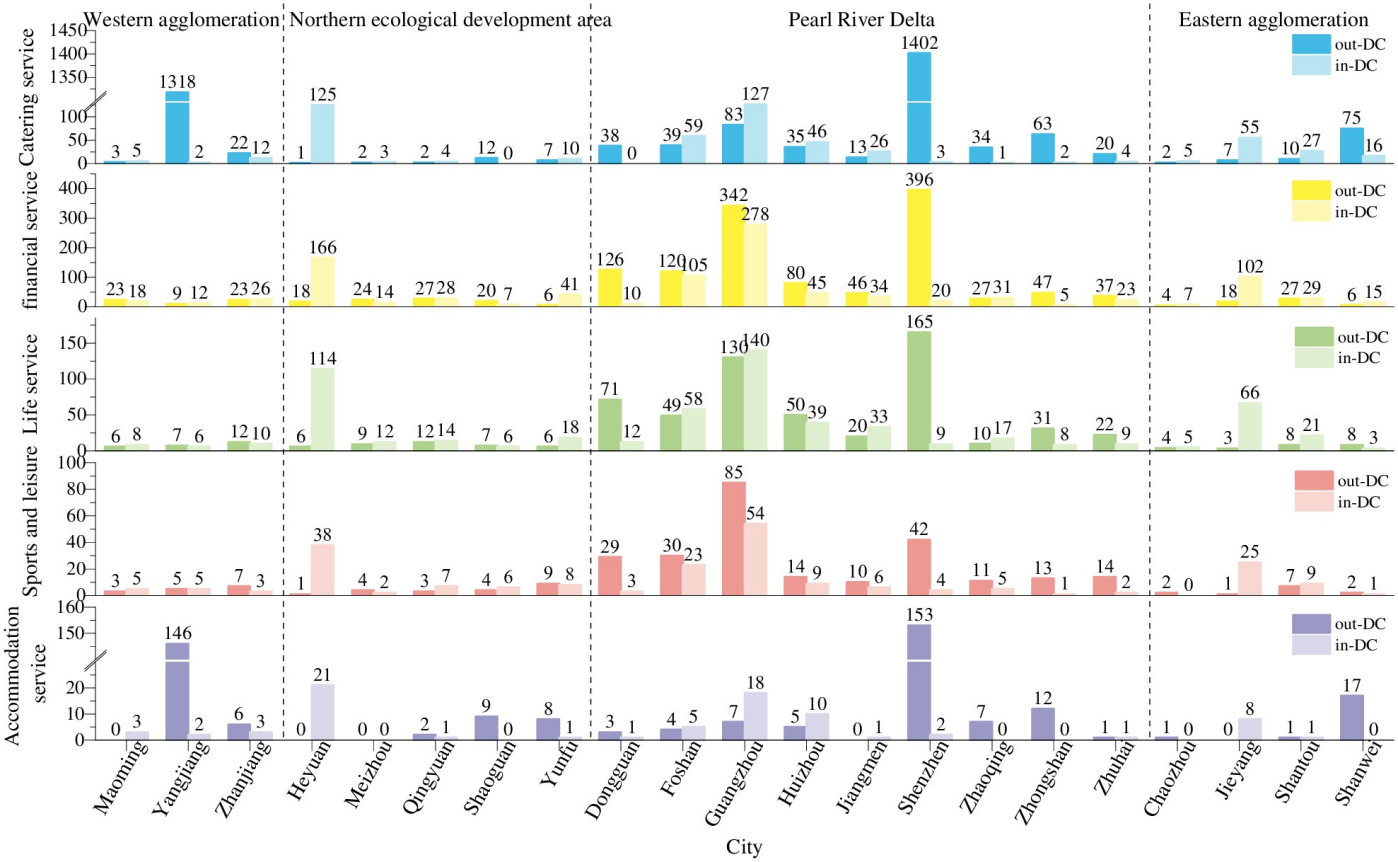
DC of corporate branches in cities of Guangdong Province.

**Fig 4:**
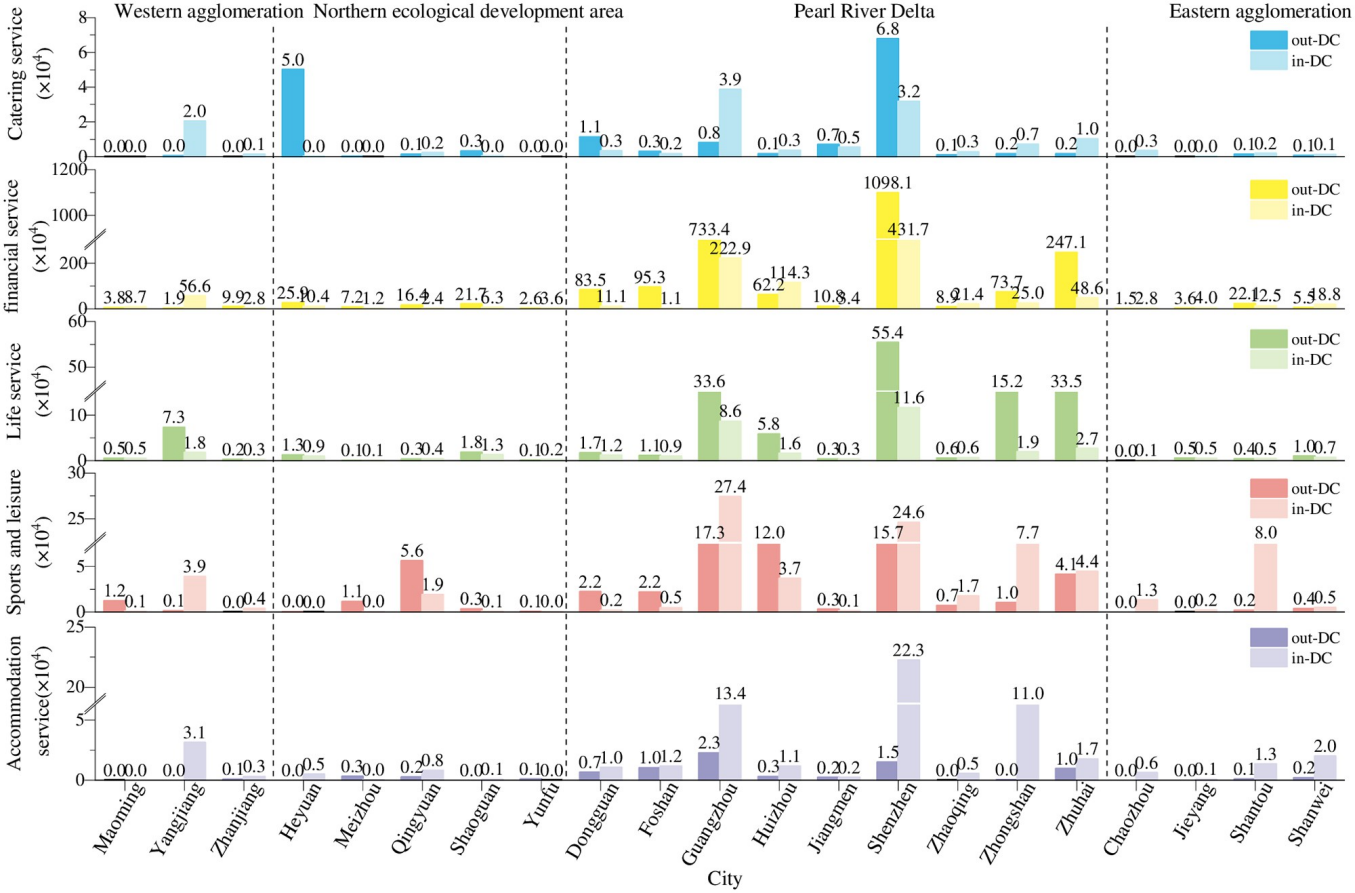
DC of corporate investments in cities of Guangdong Province.

The measurement results of the DC of the corporate branch network and corporate investment network of urban agglomerations in Guangdong are shown in Figs 3 and 4. This paper focuses on the ranking of the measurement results. Overall, with the core urban agglomeration of the Pearl River Delta as the exchange center, the other 3 urban agglomerations account for only a few exchanges in the whole province. **(1) In the western urban agglomeration**, there is a lack of regional core cities that can take on the urban functions of financial services, financial services, and sports and leisure services. More capital flow from Shantou, Shenzhen, and Guangzhou input, can reach 30776×10^4^ million yuan, and only a small amount of capital flow to Jiangmen, Zhuhai, and Guangzhou, amounting to an inflow of 3.30%. Yangjiang plays the most prominent role. Its DC of accommodation service (146) and catering service (1318) are much higher than those of Maoming and Zhanjiang. The prominence of Yangjiang’s catering service is due to the larger amount and higher number of streams; the amount of the accommodation service is low, but the number of outflows is high. This city has attracted more enterprises to set up branches, and there are more capital exchanges in catering service, financial service (out-DC of corporate investments is 56.6×10^4^, similarly below), sports and leisure (3.9×10^4^) and accommodation service (3.1×10^4^), thus making great contributions to regional economic development. **(2) In the Northern Ecological Development Zone**, the region can combine its ecological resources to attract outside tourists and provide them with tourism services, which in turn will improve the connectivity of urban networks. Heyuan’s in-DC values of catering services (125), financial services (166), life services (114), sports and leisure (38) and accommodation services (21) are much higher than nearby cities. Thus, it has attracted more foreign enterprises and amount of foreign investment in catering services, but none of the 5 cities in this region show an obvious radiating effect. The capital exchange in the Northern Ecological Development Zone is relatively singular and unstable, most of the DC values of these cities are 0, and there is almost no communication with the outside world. **In the Pearl River Delta urban agglomeration**, it must reach out to cities on the periphery of the urban network and lead them to develop together. This region is the core of the province. Guangzhou and Shenzhen have obvious attraction and radiation qualities in terms of corporate institutions and capital exchanges. Guangzhou has the largest exchange of branches. Among the 5 types, the out-DC of catering services, life services, and accommodation services are far greater than the in-DC, showing a strong radiation engine. Shenzhen is a rising star, and with its attractiveness to enterprises, it begun to catch up with and surpass Guangzhou. The DC of catering service (1402), life services (165), financial services (396) and accommodation services (153) is greater than that in Guangzhou. **In the eastern urban agglomeration**, compared to other regions, it is still lagging behind in the development of urban networks, and the network links between cities are weak. The 5 types have established branches in Jieyang. Moreover, Shanwei has a certain attractiveness for catering services and accommodation services, but the capital exchange is still scarce.

Vertically, in terms of type, the order of complexity of enterprise investment and branch network is as follows: financial service, life service, sports and leisure, catering service, and accommodation service. The measurement results of the DC of the branches of the 5 types show that Shenzhen’s catering service (1402) and financial service (396) are the highest, and the peaks of the other 3 types are small. For most cities, the corporate branch network of catering services and financial services is more complex and stable. The financial service’s DC value of corporate capital exchange far exceeds that of the other 4 types, and each city participates in the network communication. Financial services are followed by life services, catering services, sports and leisure and accommodation services. Many cities do not even have contact with the outside world, and these cities are still scattered around the periphery, such as Maoming, Meizhou, Yunfu and Jieyang.

#### 4.1.2 Characteristics of betweenness centrality

**Fig 5:**
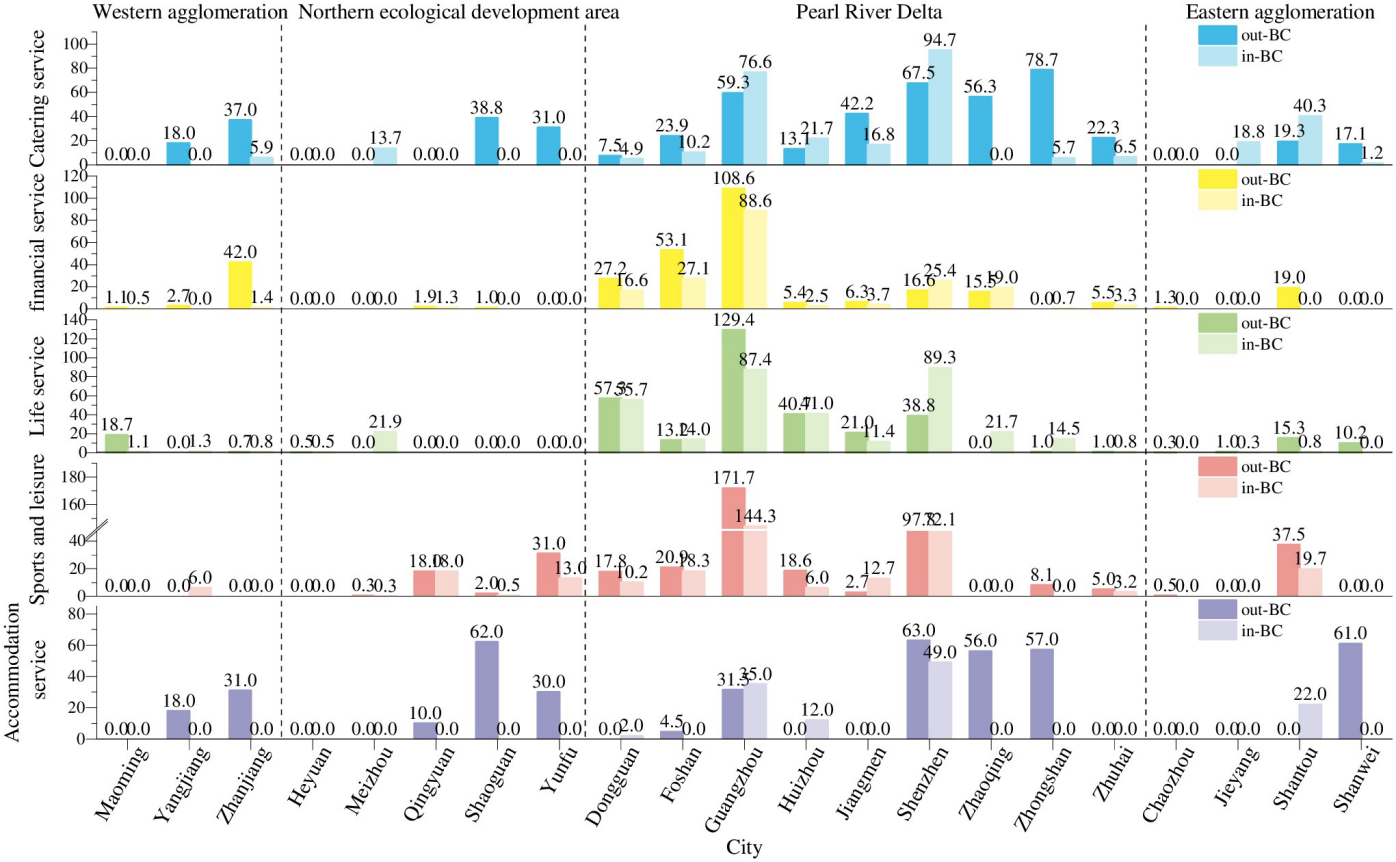
BC of corporate branches in cities of Guangdong Province.

**Fig 6:**
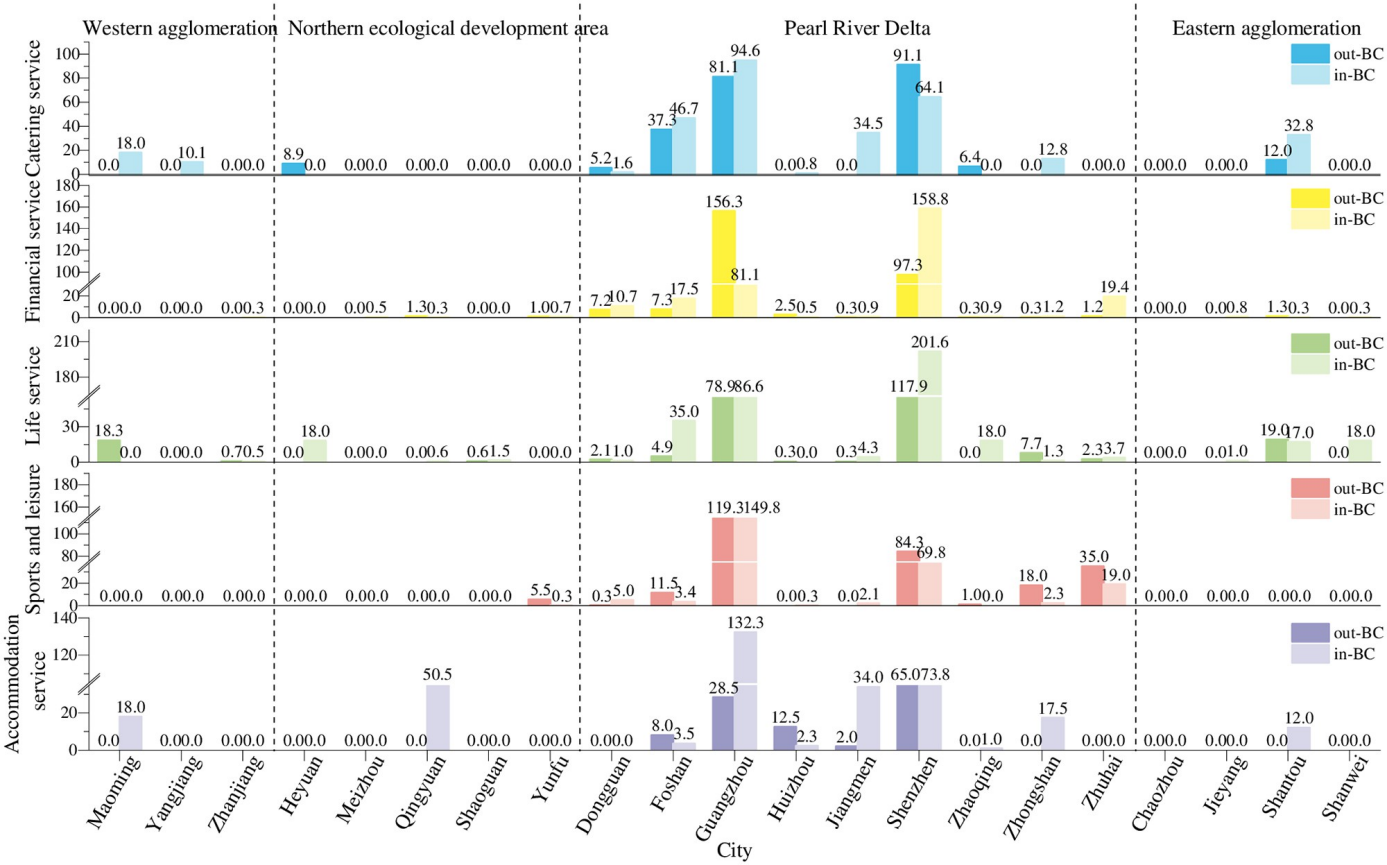
BC of corporate investments in cities of Guangdong Province.

The measurement results of the BC of 21 cities are shown in Figs 5 and 6. The distribution of intermediary cities is more balanced in the corporate branch network than in the corporate investment network. The largest difference between branches and investments is that most regional economic exchanges pass through Guangzhou and Shenzhen. Obviously, the intermediary cities are extremely concentrated, reflecting the great importance of the urban functions of Guangzhou and Shenzhen. The multicore intermediary role is more conducive to taking the development of urban networks than the single-core role. Shenzhen has also begun to undertake the important task of promoting regional development, effectively relieving the pressure on Guangzhou.

The intermediary role of financial services, life services and sports and leisure in the corporate branch network is reflected in a few cities. In addition to cities in the core area of the Pearl River Delta, many cities in the western, eastern and northern regions have a certain intermediary ability. These cities include Maoming and Shantou, which have a certain intermediary role in the 3 types. When the Pearl River Delta urban agglomeration have limited radiating capacity, cities closer to Maoming and Shantou can reach out to more resources in other cities by contacting them. Western and eastern urban agglomerations need to be developed, and regional core cities need to be developed in the Northern Ecological Development Zone.

#### 4.1.3 Characteristics of the network density

The density of the corporate branch network in Guangdong is shown in Table 3. The network connection is stronger for financial service (in-density value is 0.25, out-density value is 0.26, similarly below) and life service (0.21, 0.25) and weaker for accommodation service (0.06, 0.06). In the corporate investment of cities, the density of the 5 types is low, with the density of financial service (0.22, 0.27) being the largest, the density value of the other types is less than 0.200. This indicates that the connections among many cities in the province are weak or nonexistent. Thus, the connections among cities in the province need to be strengthened.

**Table 3:** Overall network analysis of corporates in cities of Guangdong Urban Agglomeration.

category	type	out-density	in-density	out-DCZ(%)	in-DCZ(%)	out-BC(%)	in-BC(%)
Corporate branch	Catering service	0.1476	0.15	48.42	42.11	14.74	22
	Sports and leisure	0.1476	0.1333	66.58	53.68	41.77	35.6
	Financial service	0.2548	0.2595	64.21	65.26	25.97	21.97
	Life service	0.2119	0.25	67.63	64.47	31.16	19.91
	Accommodation service	0.0571	0.0571	32.11	49.21	11.83	11.96
Corporate investment	Catering services	0.1214	0.181	49.21	52.37	21.99	21.99
	Sports and leisure	0.1333	0.1976	65.79	79.47	29.36	38.09
	Financial service	0.2214	0.2714	71.58	70	15.63	40
	Life service	0.1643	0.1786	71.58	74.47	29.25	50.34
	Accommodation service	0.069	0.131	42.11	63.16	16.43	32.03

The overall network DCZ results of the urban agglomerations of Guangdong are shown in Table 3. Regarding the DCZ of the corporate investment network, the total DCZ of the input and output of the 5 types is more than 50% and that of financial and life services is more than 70%. The in-DCZ of the 5 types is significantly greater than the out-intermediary DCZ, and the number of nodes with higher status in the capital in-network is far greater than that in the in-network. In terms of corporate branch network DCZ, the 5 types have high overall out-degree and in-DCZ in addition to accommodation service, and the network nodes have a solid ability to contact the outside world. The DCZ value of the export and entry of sports and leisure, financial services and life services is 53.68% ~ 67.63%. The overall connectivity of network city nodes is strong, and the out-corporate connectivity of sports and leisure (in-DCZ value is 53.68%, out-DCZ value is 66.58%, similarly below) and life service (64.47%, 67.63%) is greater than the in-corporate connectivity. In terms of network node intermediary ability, many number of city nodes play an intermediary ability and control role in the 5 types.

### 4.2 Analysis of the factors influencing the urban agglomeration network

To make the results more convincing, the indexes in the regression model are tested based on multicollinearity and sequential autocorrelation. The test results show that the VIF is less than 10, indicating that there is no multicollinearity. To eliminate the problem of heteroskedasticity, the standard error is used for estimation. Finally, a stepwise regression is performed to make the regression results more robust, and the final results are compared to the results of Model 3.

#### 4.2.1 The factors influencing headquarters and branches

The results of the factors influencing of branches are shown in Table 4. Overall, among the 6 factors influencing origin cities in Model 3, the P value of 3 indicators reaches the 0.01 significance level, the P value of one indicator reaches the 0.05 significance level, and all of them show a positive correlation. Six of the 9 destination city indicators are correlated, of which the P value of two indicators reaches the significance level of 0.01 and that of two indicators reaches the significance level of 0.05. Compared to the factors influencing of corporate investments, there are more relevant indicators.

**Table 4:** Regression results of corporate branches.

variable	model 1	model 2	model 3
Source city vitality			
Urban development level	0.054***		0.071***
Urban market demand	-0.001		0.001**
Reserve of urban technicians	0.141***		0.125***
Urban education resource reserve	1.286		0.819
City scale	0.001***		0.001***
Urban opening level	-0.007		-0.002
Destination city attractiveness			
Urban development level		-0.004	0.038**
Urban enterprise vitality		0.717	0.926
Urban administrative capacity		0.001	-0.452**
Reserve of urban technicians		0.015	0.061
Urban opening level		-0.005	0.016*
Urban education resource reserve		-12.255*	-14.546***
Urban science and technology support		0.006*	0.004***
City scale		0.001	-0.001
Urban market demand		-0.001*	-0.001*
Geographical proximity			
Geographical proximity	0.882***	1.281***	0.731***
Intercept	-0.027***	0.894***	0.577***
Sample size	376	376	376

Note:

*: *P*<0.10;

**: *P*<0.05;

***: *P*<0.01

In terms of the origin city vitality: in Model 3, there is a significant positive correlation between the urban development level (P = 0.071***) and urban market demand (P = 0.001**), which shows that the urban development level has a great impact on the location selection of headquarters. The higher the level of urban economic development is, the more the area will provide solid capital support for the establishment and stable development of headquarters, thus providing it with enough power to support enterprises in establishing branches abroad. The correlation coefficient of urban demand is small, indicating that its impact is negligible. There is a significant positive correlation for the reserve of urban technicians (P = 0.125***), but there is no such index in the factors influencing the attractiveness of destination cities, which shows that the reserve of technicians has a greater impact on enterprises. In terms of the flow of branches, a high-quality reserve of headquarters technicians is also the primary factor for the outward expansion of enterprises. City scale (P = 0.001***) also positively impacts the establishment of headquarters. Compared to smaller cities, more headquarters have better urban infrastructure, greater market demand, and more resources to promote corporate development in medium to large cities. Therefore, more headquarters are established in cities with higher development levels, such as Guangzhou, Shenzhen, and Dongguan. This influencing factor is recessive. The reserve of educational resources and the degree of urban openness have no significant impact on establishing headquarters and branches.

In terms of destination city attractiveness, according to the arrangement, 6 indicators have reached the significance level, and only 3 indicators in Model 2 have P-values reaching the significance level of 0.1. This shows that the influential factors cannot be effectively found by considering only the impact indicators of destination cities alone. At the same time, it shows that the model in this paper has certain advantages. The urban development level (P = 0.038**) shows a significant positive correlation, indicating that cities with a higher economic level are more attractive to the establishment of branches, and the establishment of branches in such cities will receive more financial support, which is conducive to the overall development of enterprises. There is a significant positive correlation between urban science and technology support (P = 0.004***). The degree of urban science and technology support breakthroughs can show a city’s attitude toward supporting corporate development. The more technology expenditure funds there are the greater the city’s capacity for the number and space of enterprises. The urban administrative capacity (P = -0.452**), urban educational resource reserve (P = -14.546***), and urban market demand (P = -0.001*) are negatively correlated. The regression coefficient of urban educational resource reserves is -14.546 (P < 0.01) in Model 3, which indicates a great impact. This shows that branches tend to be established in areas with fewer educational resource reserves, while regional headquarters are more concentrated in areas with more educational resource reserves. The coefficient of urban administrative capacity is also large, which is consistent with the former finding. Branches tend to be established in general cities, while headquarters tend to be established in the region’s central city. The annual average population coefficient is close to 0, indicating that urban demand has a positive but small impact on branch establishment.

Moreover, geographical proximity: geographical proximity shows a significant positive correlation in all three models, indicating that the stronger the geographical proximity of the city is, the more branches tend to be set up there, which is still in line with the cognition of location theory.

#### 4.2.2 The factors influencing of corporate mutual investment

The regression results of the factors influencing of corporate investments are shown in Table 5. The P value of 9 of the 13 indicators reaches a significance level of 0.01, indicating that the capital exchange network of enterprises is affected by many factors.

**Table 5:** Regression results of corporate investments.

variable	Model 1	Model 2	Model 3
Source city vitality			
Urban development level	0.154***		0.214***
Urban enterprise vitality	-0.045**		-0.033*
City scale	0.002***		0.001***
Urban education resource reserve	2.162		-23.588***
Urban market demand	0.002***		0.002
Destination city attractiveness			
Urban development level		-0.090**	0.076**
Urban enterprise vitality		-0.035*	-0.051
Urban administrative capacity		0.195	-0.301***
Reserve of urban technicians		0.005	0.034***
Urban opening level		0.001*	0.001
Urban science and technology support		0.001	-0.001***
Urban market demand		0.003***	-0.001***
Geographical proximity			
Geographical proximity	0.944***	1.947***	0.896***
Intercept	6.795***	6.598***	5.872***
Sample size	352	352	352

Note:

*: *P*<0.10;

**: *P*<0.05;

***: *P*<0.01

In terms of origin city vitality, the coefficient of the urban development level (P = 0.214***) is significantly positive, indicating that the higher the urban development level of the local city is, the stronger the endogenous power of the city and the more capable it is of supporting the external capital exchange of enterprises. At the same time, the cross-regional capital exchange of enterprises also needs to occur on a larger scale. The urban scale (P = 0.001**) coefficient is significantly positive, but the coefficient is small. This indicates that the larger the urban scale and the higher the degree of infrastructure improvement are, the more local enterprises can exchange funds across regions. However, the coefficient is small, indicating that the degree of impact is also small. Urban scale and infrastructure improvement is a long-term process, and its impact is implicit and indirect. The index of urban corporate vitality (P = -0.033*) has a negative correlation. The correlation is general and the coefficient is small, which indicates that urban corporate vitality will solidify its ability to connect external capital to suit the current market, local resources and local technology. In Model 3, the P value of the educational resource reserve is -23.588***, with a significant negative correlation. The higher the educational resource reserve is in local cities, the more the capital exchange of enterprises will be limited. Notably, the correlation coefficient of the urban educational resource reserve is positive in Model 1 and has no impact on the corporate capital network, but after the factors influencing foreign exchange cities are added, the coefficient is significantly negative. This indicates that the impact of educational resource reserve on the capital exchange between enterprises in cities is affected by the attractiveness of foreign exchange cities. Thus, it is necessary to conduct a comprehensive analysis of the whole (origin city, destination city and geographical proximity of capital flow) to obtain practical results. While urban demand is significantly positively correlated in Model 1, the coefficient in Model 3 is 0.002, which is not significant, so it will not affect the capital exchange of enterprises.

In terms of destination city attractiveness, the P values of the 4 indicators in Model 3 reach a significance level of 0.01, and the P value of one indicator reaches a significance level of 0.05, indicating that the attributes of destination cities have a significant impact on corporate capital exchange. However, the urban administrative level, scientific and technological personnel reserves and scientific and technological expenditures have no significant impact on corporate capital exchange in Model 2. After the indicators of origin cities are added, the urban development level (P = 0.076**) shows a significant positive correlation. This indicates that more capital exchanges between enterprises flow to more economically developed areas in search of richer technological progress, personnel reserves and corporate resources and broaden the market. Of course, more economically developed cities need to gather more foreign funds to inject impetus into their own economic development power. The reserve of urban scientific and technical (P = 0.034***) is also significantly positively correlated. The more scientific and technical personnel, technical services and geological exploration personnel a city has, the more likely it is to have sufficient capital and talent to meet the needs of foreign enterprises in terms of technological innovation and project cooperation. Furthermore, urban administrative capacity (P = -0.301***), urban science and technology support (P = -0.001***) and urban market demand (P = -0.001***) are significantly negative. This indicates that the vast majority of foreign capital exchange of corporate funds in urban agglomerations of Guangdong flows from provincial capital cities, subprovincial cities and special economic zones to other cities, while less capital flows from general cities to regional central cities. The actual amount of foreign capital used has no impact on the overall level.

In terms of geographical proximity: the P values in the 3 models are significant at the 0.01 level, and the coefficients are more than 0.8, indicating that the space-time distance between cities has a strong impact on capital exchange. The closer the space-time distance is, the greater the capital flow between cities, and the farther the distance is, the greater the transportation cost, which greatly hinders the capital network exchange between cities.

## 5. Conclusion

1. Among the 21 cities, more urban nodes have high out-DCZ and in-DCZ, strong control ability and intermediary ability. The 5 major types of enterprises in the province show extremely significant centralized characteristics, with Shenzhen and Guangzhou as the primary centers. Especially in the calculation results of the BC of the corporate investment network, the values of catering service, finance service, life service, sports and leisure, and accommodation service are far higher than those of other cities, and other cities in the Pearl River Delta are secondary power centers.
2. Financial service networks are the most complex and have stable secondary networks, The out-density and in-density of their corporate branches are greater than 0.25, and the density of their corporate investment network is greater than 0.22, far higher than that of the other 4 types. These are followed by life service networks. which, along with accommodation networks, are the simplest and sparsest.
3. Among all kinds of networks, the measurement results are in line with the development of Guangdong. The Pearl River Delta urban agglomeration is forming a regional dual-engine radiation center. Guangzhou and Shenzhen still have the highest status. Yangjiang, Heyuan, Jieyang and Shanwei, which rank prominently in other urban agglomerations, especially Yangjiang and Heyuan are geographically close to the core urban agglomeration of the Pearl River Delta, and except for Heyuan, other cities are all in the Coastal Economic Belt, which makes a great contribution to the coordinated development of their urban agglomeration.
4. The construction of the model requires the joint participation of the factors influencing of the origin and destination to identify the influential factors effectively. Gradually adding the factors influencing of origin, destination and geographical proximity to the regression indicates that some factors (e.g. urban development level, urban education resource reserve, urban market demand) do not contribute much to a single model but significantly affect the whole model. The reason is that some factors influencing of the urban network are jointly constrained by the origin and destination cities.
5. Geographical proximity and urban development level have a lasting and strong impact on the urban network. Urban administrative capacity shows a very significant negative correlation in the urban network, indicating that more enterprises are satisfied with the existing market and resources and have fewer external contacts. In the corporate investment network, there is a very significant negative correlation between the educational resource reserve of the origin city and the capital flow of the city, which reflects the problem of insufficient external radiation capacity of the city. The most significant problem of the corporate network is that cities with higher economic development levels and stronger administrative ability still have weak outward radiation.

## 6. Discussion

1. Reliability of methods: The research method of network centrality comprises a set of methods specially used for urban networks. Since the rise of urban networks, this method has been verified, improved and universal. The urban network influence model, which is constructed with 3 indicators: origin city vitality, destination city attraction and geographical location, has made some improvements on the spatial interaction model that was more widely used before. Through the results of this progressive model, it can also be seen that considering the vitality of the origin city and the destination city at the same time is more conducive to finding the factors that affect the urban network. Therefore, the two methods used in this paper are reliable and can be applied to other flow space networks that are similar to urban networks, which have 3 elements: origin, destination and distance.
2. Policy recommendations: 1) The connectivity of the Pearl River Delta urban agglomeration with other cities should be strengthened. According to the results of factor analysis in this paper, these cities with higher economic development levels and stronger administrative ability need to strengthen their external radiation ability. 2) Priority should be given to the stable development of a secondary network of headquarters and branches in the western urban agglomeration of Guangdong. Specifically, the government should promote the development of the secondary networks of finance, life service, sports and leisure in the western urban agglomeration of Guangdong, while maintaining the stability of the secondary networks of catering and accommodation in the ecological development areas of western, eastern and northern Guangdong. 3) The development of the urban capital exchange subnetworks in the urban agglomerations in eastern Guangdong and the eco-development areas in northern Guangdong should progress. It is obvious that the secondary networks of catering, financial and lifestyle services have yet to be formed in the eastern urban agglomeration of Guangdong, and the secondary networks of financial, lifestyle and accommodation services in the Northern Ecological Development Zone also have yet to be formed. 4) Investment in education resource reserves for the city should be increased to pave the way for attracting more inward capital.
3. Study limitations: The connection between the study area and the outside world needs to be considered. In the context of the global urban network, each urban network studied is not isolated but is a part of the global urban network. However, due to the data limitations, this paper can only examine the study area as an independent system.
4. Discussion of flow space: The urban network of Guangdong has shown obvious regional cluster characteristics. The urban agglomeration in the Pearl River Delta has an obvious driving effect on the better-located cities nearby, effectively reducing the space-time distance from nearby coastal cities. In addition, the coastal city network has gradually developed into a trend of regional agglomeration. It can be seen that if the urban network can reduce the distance cost, it can completely move from one center to many centers, and finally the entire urban network can be unblocked. Driven by the Pearl River Delta urban agglomeration, the urban network of the Northern Ecological Development Zone is also very fragile, so the urban networks of other general regions still need to be developed.
5. Future research prospects: The city node has a unique position as the initiator of the connection in the network. Research on the spatial structure of the urban node can inform a deeper understanding of urban networks. The urban center is the urban functional core with highly intensive land use and intensive economic activities in the nodes of the urban network. The study of the vitality of the functional area of the urban center can explain the deep mechanism of the formation of the urban network.

## References

[1] Camagni R, Salone C. Network Urban Structures in Northern Italy: Elements for a Theoretical Framework. Urban Stud. 1993;30(6):1053–64. doi: 10.1080/00420989320080941

[2] Smith DA, Timberlake M. Conceptualising and Mapping the Structure of the World System’s City System. Urban Stud. 1995;32(2):287–304. doi: 10.1080/00420989550013086

[3] Hu YX, Xiang WC, Huang J, Gao X, Zhang ZC, Wang M, et al. Towards 6G wireless communication networks:vision, enabling technologies, and new paradigm shifts. Science China (Information Sciences). 2021;64(01):5–78.

[4] Shen LZ, Gu CL. Integration of Regional Space of Flows and Construction of Global Urban Network. Scientia Geographica Sinica. 2009;29(6):787–93. doi: 10.1109/CLEOE-EQEC.2009.5194697

[5] Chrisanthi Avgerou. The Informational City: Information Technology Economic Restructuring and the Urban Regional Process. Eur J Inform Syst. 1991;1(1):76–7. doi: 10.1057/ejis.1991.11

[6] Moss ML, Townsend AM. The Role of the Real City in Cyberspace: Understanding Regional Variations in Internet Accessibility. In: Janelle DG, Hodge DC, editors. Information, Place, and Cyberspace: Issues in Accessibility. Berlin, Heidelberg: Springer Berlin Heidelberg; 2000. p. 171–86.

[7] Akhavan M, Ghiara H, Mariotti I, Sillig C. Logistics global network connectivity and its determinants. A European City network analysis. J Transp Geogr. 2020;82:102624. doi: 10.1016/j.jtrangeo.2019.102624

[8] Luis I, Harald Z. The metabolic urban network: Urbanisation as hierarchically ordered space of flows. Cities. 2020;109(prepublish).

[9] Zhang P, Zhao Y, Zhu X, Cai Z, Shi S. Spatial structure of urban agglomeration under the impact of high-speed railway construction: Based on the social network analysis. Sustain Cities Soc. 2020;62:102404. doi: 10.1016/j.scs.2020.102404

[10] Yu H, Yang J, Li T, Jin Y, Sun D. Morphological and functional polycentric structure assessment of megacity: An integrated approach with spatial distribution and interaction. Sustain Cities Soc. 2022;80:103800. doi: 10.1016/j.scs.2022.103800

[11] Zhang DH, Zhou CS, Sun DQ, Qian Y. The influence of the spatial pattern of urban road networks on the quality of business environments: the case of Dalian City. Environment, Development and Sustainability. 2022;24(7):9429–46.

[12] Yu WB, Yang J, Sun DQ, Yu HS, Yao Y. Spatial-Temporal Patterns of Network Structure of Human Settlements Competitiveness in Resource-Based Urban Agglomerations. Front Env Sci-Switz. 2022;10: 9429–46. doi: 10.3389/fenvs.2022.893876

[13] Zhang DH, Zhou CS, He BJ. Spatial and temporal heterogeneity of urban land area and PM2.5 concentration in China. Urban Clim. 2022;45:101268. doi: 10.1016/j.uclim.2022.101268

[14] David B, Laura G, Reijer H, Deborah L, Maëlys W. Unpacking the advanced producer services complex in world cities: Charting professional networks, localisation economies and markets. Urban Stud. 2021;58(6). doi: 10.1177/0042098020908715

[15] Short JR, Kim YH, Kuus M, Wells H. The Dirty Little Secret of World Cities Research: Data Problems in Comparative Analysis. International Journal of Urban & Regional Research. 2010;20(4):697–717. doi: 10.1111/j.1468-2427.1996.tb00343.x

[16] Taylor PJ, Catalano G, Walker DRF. Measurement of the World City Network. Urban Stud. 2002;39(13): 2367–76. doi: 10.1080/0042098022000027013

[17] Neal Z. Differentiating Centrality and Power in the World City Network. Urban Stud. 2011;13(48):2733–48. doi: 10.1177/0042098010388954

[18] Yang X, Derudder B, Taylor PJ, Ni P, Shen W. Asymmetric global network connectivities in the world city network, 2013. Cities. 2017. doi: 10.1016/j.cities.2016.08.009

[19] Blumenfeld-Lieberthal E, Portugali J. Network Cities: A Complexity-Network Approach to Urban Dynamics and Development. geojournal library. 2010. doi: 10.1007/978-90-481-8572-6_5

[20] Alderson S, Beckfield J. Power and Position in the World City System. Am J Sociol. 2004;109(4):811–51. doi: 10.1086/378930

[21] Smith DA, TimerlankI MF. World City Networks and Hierarchies, 1977–1997: An Empirical Analysis of Global Air Travel Links. Am Behav Sci. 2001;44(10):1656. doi: 10.1177/00027640121958104

[22] Zhou YX, Yong HZ. Looking into the network structure of Chinese urban system from the perspective of air. Geogr Res-Aust. 2002(03):276–286. (in Chinese)

[23] Ducruet C, Cuyala S, Hosni AE. Maritime networks as systems of cities: The long-term interdependencies between global shipping flows and urban development (1890–2010). J Transp Geogr. 2018;66(jan.):340–55. doi: 10.1016/j.jtrangeo.2017.10.019

[24] Chen W, Liu W, Ke W, Wang N. Understanding spatial structures and organizational patterns of city networks in China: A highway passenger flow perspective. J Geogr Sci. 2018;28(4). doi: 10.1007/s11442-018-1485-x

[25] Bonnetain L, Furno A, El Faouzi N, Fiore M, Stanica R, Smoreda Z, et al. TRANSIT: Fine-grained human mobility trajectory inference at scale with mobile network signaling data. Transportation Research Part C: Emerging Technologies. 2021;130:103257. doi: 10.1016/j.trc.2021.103257

[26] Huang X, Zhang L, Ding Y. The Baidu Index: Uses in predicting tourism flows—A case study of the Forbidden City. Tourism Manage. 2017;58(FEB.):301–6. doi: 10.1016/j.tourman.2016.03.015

[27] Hall P, Pain K. The Polycentric Metropolis: Learning from Mega-City Regions in Europe. Econ Geogr. 2010;86(3):323–4. doi: 10.1111/j.1944-8287.2010.01080.x

[28] Sun Y, Shao Y, Chan E. Co-visitation network in tourism-driven peri-urban area based on social media analytics: A case study in Shenzhen, China. Landscape Urban Plan. 2020;204(1):103934. doi: 10.1016/j.landurbplan.2020.103934

[29] Zhang S, Wang X. Does innovative city construction improve the industry–university–research knowledge flow in urban China? Technol Forecast Soc. 2022;174:121200. doi: 10.1016/j.techfore.2021.121200

[30] Rui Y, Tong C, Fujun L. The Impacts of production linkages on cross-regional collaborative innovations: The role of inter-regional network capital. Technological Forecasting & Social Change. 2021;170. doi: 10.1016/j.techfore.2021.120905

[31] Gan C, Voda M, Wang K, Chen L, Ye J. Spatial network structure of the tourism economy in urban agglomeration: A social network analysis. J Hosp Tour Manag. 2021;47(6):124–33. doi: 10.1016/j.jhtm.2021.03.009

[32] Castellanos González ME, Miranda Vera CE, Marianela Morales C, García Dueñas R, Moreira González ÁR, León Pérez ÁR, et al. Social knowledge networks for promoting environmental education in coastal communities from central-southern region of Cuba. Reg Stud Mar Sci. 2020;35. doi: 10.1016/j.rsma.2020.101115

[33] Jia J, Liang X, Ma G. Political hierarchy and regional economic development: Evidence from a spatial discontinuity in China. J Public Econ. 2021;194:104352. doi: 10.1016/j.jpubeco.2020.104352

