# Correction: Assessment of network structure characteristics and factors of corporate flows in Guangdong Province

**DOI:** 10.1371/journal.pone.0304246

**Published:** 2024-05-17

**Authors:** Xuejiao Chen, Yong He, Teng Long, Junxiu Wang, Xueye Chen

The following information is missing from the funding section: This study was supported by the Open Fund of Key Laboratory of Urban Land Resources Monitoring and Simulation, Ministry of Natural Resources (KF202207012).
